# Book Review: The Models of Engaged Learning and Teaching Connecting Sophisticated Thinking From Early Childhood to PhD

**DOI:** 10.3389/fpsyg.2021.750854

**Published:** 2021-08-24

**Authors:** Qianyu Cao

**Affiliations:** School of Foreign Languages, Chengdu University of Information Technology, Chengdu, China

**Keywords:** book review, engaged learning, teaching thinking, constructivist learning, education

The prime focus of John Willison's innovative monograph, entitled *The Models of Engaged Learning and Teaching Connecting Sophisticated Thinking from Early Childhood to PhD*, is to assist learners to learn efficaciously to resolve intricate problems, contemplate innovatively and critically, and finally make practical decisions. In so doing, the book foregrounds the quintessential components of the Models of Engaged Learning and Teaching (MELT) to enrich our understanding of the dynamic interplay across multifarious educational contexts, postulations, and tasks by enabling a myriad of viewpoints and practices to work concomitantly to boost the enhancement of students' sophisticated thinking. Furthermore, the MELT illuminate the viable ways through which educational energies and ideas can work together to get rid of the vicious circle of our solutions that contribute to more problems. Representing diverse perspectives of education theory and practice and reconciling them, the MELT embarks on discovery learning, objectivist learning, and constructivist learning from primary school to Ph.D. studies across all disciplines. In other words, the essence of the book is echoed as “the book introduces the MELT as a way to conceptualize how such enabling, connecting, deepening and engaging may take place” (p. 9).

The book is divided into seven chapters. Chapter 1 conceptualizes the MELT, explicates their objectives, and provides an overview of the MELT's six facets of sophisticated thinking that is detailed along a continuum of learning autonomy. Besides, this chapter elaborates on different modules of learning that are rampant across formal education and diverse disciplinary and interdisciplinary contexts by highlighting the dire need to enhance sophisticated thinking so that we can address educational and planet-wide problems provided that we can (re)conceptualize models such as MELT to integrate disparate teaching approaches and ideas. Chapter 1 also outlines the structure of the book, delineating the components that are essential to each MELT facet and to learning autonomy. The author cogently argues that the facets of MELT are inextricably bound processes whose descriptions function as triggers and connectors, so these six facets cannot independently function properly to reflect the meaning for every context. Therefore, their practicality is highly contingent on educators who capitalize on them and who know what needs to take place in any learning context that they are fostering. Finally, this chapter clearly justifies the need to draw on a wide range of teaching and learning strategies that are definitely crucial to effective engagement, hoping that these conceptions are concomitantly taken into consideration as a holistic package to be more mutually supportive.

The title of Chapter 2 is “What Will We Use” that aims to represent a thorough explication of the MELT and its six facets of sophisticated thinking, by paying close attention to how much support students need with respect to *learning autonomy* from primary school to university. Moreover, the author appraises the extant literature that has informed the development of MELT, articulating that much of the background is descriptive and lacks theoretical underpinnings and evidence-based studies. Providing several instances of teachers utilizing MELT, Chapter 3 showcases how teachers from primary school to postgraduate education can arrange and facilitate more sophisticated thinking across the educational trajectory by sharing their own experiences and pedagogical interpretations of what constructs MELT and what should be implemented in each of these educational contexts.

Chapter 4 brings together the competing theories, namely Objectivism, Social Constructivism, and Personal Constructivism, which underpin the MELT, in productive tension. More specifically, the author adroitly links these competing theoretical underpinnings to “the *learning autonomy continuum*, with the aim of arousing awareness and choice of where to operate on or across this continuum” (p. 34–35). Chapter 5 justifies the need to take into account the seminal and contemporary learning theories such as Threshold Concepts, Cognitive Load Theory, Connectivism, and Reflective Practitioner, by considering what they mean for recent educational practices in light of MELT. The author unpacks and situates these learning theories on MELT's *continuum of learning autonomy*, and empathizes that it is pivotal that teachers are cognizant of these theories and their application in action research and classroom contexts.

Chapter 6 delves into the relationship between humans and the environment and problematizes why things happened in a way that inevitably culminated in environmental devastation and social upheaval. The author hopefully and cogently argues that in order to alleviate the existing problems and terminate the vicious circle of the problems, MELT may be part of a solution that does not lead to more problems. Chapter 7 deals with how much scaffolding and guidance students need while utilizing MELT's learning autonomy continuum. This chapter takes into account those individuals who are engaged in education and the amount of scaffolding they need to put into practice MELT in diverse educational settings. The author contends that autonomy in MELT is a relational word, which is bound to ownership of teaching and learning, so the need for ownership and empowerment seems to play a crucial role when considering what kind of scaffolding is required by students and teachers. The interconnected and coherent trajectory of students' learning highly depends on teachers, parents, schools, and universities; therefore, to develop ownership and empowerment, teachers need to be autonomous as well.

I personally find this book insightful and thought-provoking in that it appropriately problematizes how teacher-student relationship, engagement, and autonomy can be enhanced through the MELT by conceptualizing different models, drawing on different conceptual frameworks, and exemplifying real examples from primary to university education. Besides, each fact of the MELT is well-explicated throughout the book, paving the way for caregivers, researchers, learning advisors, learning designers, teachers, parents, practitioners, and students from primary school to Ph.D. to put them into practice. However, had the author included more evidence-based studies to delineate how these facets can be implemented, this monograph would have been more insightful.

## Author Contributions

The author confirms being the sole contributor of this work and has approved it for publication.

## Conflict of Interest

The author declares that the research was conducted in the absence of any commercial or financial relationships that could be construed as a potential conflict of interest.

## Publisher's Note

All claims expressed in this article are solely those of the authors and do not necessarily represent those of their affiliated organizations, or those of the publisher, the editors and the reviewers. Any product that may be evaluated in this article, or claim that may be made by its manufacturer, is not guaranteed or endorsed by the publisher.

